# Artificial intelligence-powered models in predicting mortality in maternal, newborn, and children under five: a systematic review protocol

**DOI:** 10.1186/s13643-026-03138-5

**Published:** 2026-03-07

**Authors:** Yingxiang Huang, Yuxuan Li, Zheyue Jia, Jingxuan Zhuge, Zhijun Ding, Yanyi Zhang, Ruoxuan Li, Kun Tang

**Affiliations:** 1 Karolinska Institutet, Solna Campus, Stockholm, Sweden; 2 Vanke School of Public Health, Tsinghua University, Beijing, China; 3 School of Basic Medical Sciences, Tsinghua University, Beijing, China; 4 Global Health Research Center, Duke Kunshan University, Kunshan, Jiangsu Province, China; 5 School of Public Health, Fudan University, Shanghai, China; 6 Chinese Association of Sexology, Hunan Province, Changsha, China; 7 Graduate School of Art and Science, Yale University, New Haven, CT USA

**Keywords:** Artificial intelligence, Pregnant women, Infant, Children under 5, Mortality

## Abstract

**Background:**

Maternal, newborn, and under-five mortality remain significant global health challenges, with 287,000 maternal deaths reported in 2020 and 4.9 million under-five deaths in 2022. The use of artificial intelligence in prediction models has shown great potential in predicting these mortalities. Artificial intelligence (AI)-powered models are capable of processing complex datasets to identify non-linear relationships and patterns, potentially outperforming traditional statistical methods in predictive accuracy, thereby enabling early detections and interventions more specifically. As AI develops rapidly, it is imperative to carry out systematic evaluations and comparisons of the accuracy, sensitivity, and applicability of these models to ensure the most efficient and effective use of AI in reducing mortality in these groups. The objective of this protocol is to outline the methods that will appraise, synthesise, and compare the existing literature in this domain.

**Methods:**

The review will follow the *Preferred Reporting Items for Systematic Reviews and Meta-Analysis 2020* guidelines, implementing a comprehensive search strategy across 14 databases, including PubMed, Embase, and Scopus. The review will refer to the most recent multiple methodological guidelines applicable to this study to determine inclusion and exclusion criteria and the content of the included literature. The articles will then be integrated, assessed, and compared for transparency, standardisation, and fairness, as well as model performance, generalisation, and heterogeneity, in accordance with the aforementioned methodological guidelines. Where possible, meta-analyses of the performance of models meeting specific criteria will also be conducted to better identify more effective models.

**Discussion:**

This protocol outlines the methodology used in a systematic review of artificial intelligence prediction models for predicting maternal, neonatal, and under-five mortality. Key anticipated challenges include comprehensively identifying bias and heterogeneity, establishing reasonable and transparent standards for quantitative synthesis, and addressing statistical dependence introduced multiple models within original studies. To address these, we will apply a structured taxonomy of bias, pre-defined tiered synthesis rules, and methods such as robust variance estimation and bivariate models. We expect that this review will fill important gaps in the field and provide insights that support the advancement of artificial intelligence in maternal and child healthcare.

**Systematic review registration:**

PROSPERO CRD42024569282.

**Supplementary Information:**

The online version contains supplementary material available at 10.1186/s13643-026-03138-5.

## Background

Despite a decline in maternal, newborn, and under-five mortality rates over the past two decades, the number of deaths remains alarmingly high. An estimated 287,000 maternal deaths occurred worldwide in 2020, along with approximately 4.9 million deaths among children under five in 2022 [1]. These mortality rates impose substantial economic costs, including heightened healthcare expenditures and lost productivity, which significantly strain healthcare systems and families [2]. A report from the *USA* estimated that the economic burden of maternal deaths in that country alone in 2019 would amount to approximately $30.8 million [3]. Indeed, the situation is even more dire in low- and middle-income countries, where gender discrimination and economic inequality exacerbate the impact of maternal and child deaths [4, 5]. According to the *World Bank*, the combined economic cost of maternal and under-five mortality in these countries can reach as much as 5% to 10% of *gross domestic product* (*GDP*) [6]. Conversely, the expected economic benefits of preventing these deaths are substantial [7]. To improve maternal and child survival and meet the *Sustainable Development Goals* (*SDGs*), women and infants must have access to better quality, more affordable, and more equitable health care before, during, and after childbirth [1, 8].

Among healthcare services that aim to reduce deaths in the specific population, the introduction of prediction models has demonstrated great potential by enabling early detection and intervention [9, 10]. Traditional prediction models typically rely on empirical scoring criteria or logistic regression, which is a foundational technique in machine learning. These methods, while effective, often involve simplifying the selection and analysis of predictors [11]. In recent years, artificial intelligence (AI)-powered prediction models, utilising advanced algorithms like neural networks and random forests, seem to outperform the traditional ones [9, 12]. Developed models based on AI excel at capturing non-linear relationships and complex patterns, automatically extracting and selecting features to enhance predictive performance, especially when larger datasets are available [11, 13]. As the field of AI advances and becomes more sophisticated, its potential to predict mortality in these vulnerable populations is significantly improving. Consequently, a growing number of studies are being conducted to further explore and validate the capabilities of AI in this domain [14–16].

However, research in this field faces significant challenges regarding complexity and heterogeneity. Although numerous models have been developed and internally validated, few have undergone rigorous external evaluation, and none has yet been widely implemented in clinical practice [9, 17]. Variability in development settings, predictor selection, and modelling approaches has created a chaotic landscape; even when a study reports superior performance for a particular algorithm, such conclusions may be biased if sources of heterogeneity are not scientifically examined [17–19].

More importantly, existing systematic reviews also have limitations; while they summarised algorithms and development procedures, they lack a definitive horizontal comparison between models, particularly between AI and traditional statistical methods [9, 13]. Moreover, these previous related reviews, published in 2021 and 2022 with narrow population scopes, lacked guidance from more advanced methodological frameworks. As a result, they could not fully assess how heterogeneity and bias influence model performance. An updated synthesis is therefore urgently needed to keep pace with the rapid evolution of this research field.

In summary, the field urgently needs to address the bias in model development and validation, identify superior algorithms, and explore model transferability to better support decisions in external validation and clinical practice. To meet this need, our review will be the first to comprehensively evaluate and compare the application patterns of AI and traditional models in predicting maternal, newborn, and under-five mortality, quantitatively synthesise model performance and performance differences, and examine how heterogeneity and bias influence these outcomes. A rigorous protocol is essential for this work. Thus, we introduce an innovative approach that integrates several state-of-the-art methodological guidelines and provides a systematic strategy for embedding them into the review process. We further propose the first workflow in this field for the quantitative synthesis and comparison of algorithmic performance. Overall, this protocol outlines the methodological plan for a systematic review aimed at determining whether AI models outperform traditional approaches in predicting mortality across maternal, newborn, and under-five populations, with the broader goal of advancing AI in healthcare and supporting sustainable development.

## Methods

To ensure the completeness and transparency of this review, the *Preferred Reporting Items for Systematic Reviews and Meta-Analysis* 2020 (*PRISMA2020*) [20, 21] and the *Transparent Reporting of Multivariable Prediction Models for Individual Prognosis or Diagnosis: Checklist for Systematic Reviews and Meta-Analyses* (*TRIPOD-SRMA*) [22] will be used as the reporting guidelines. In this protocol, the *Preferred Reporting Items for Systematic Reviews and Meta-Analysis Protocols* (*PRISMA-P*) [23, 24] is employed as the reporting checklist (Additional file 1).

### Methodological guidelines

Currently, well-established methodological guidelines are available for systematic reviews of prediction model development and/or evaluation studies. Adherence to these guidelines ensures the systematicity and comprehensiveness of this review. Specifically, these include:

1. Define research question and data extraction: *CHARMS* checklist
  The *Checklist for Critical Appraisal and Data Extraction for Systematic Reviews of Prediction Modelling Studies* (*CHARMS* checklist, Additional file 2) [25] is a tool specifically designed for systematic reviews of prediction models, providing a standardised framework to ensure comprehensiveness, systematicity, and consistency in the review process. It helps to clarify the research question and guides the description of the type and purpose of the models used. The checklist also provides a standardised data extraction tool that covers important aspects such as model variables, outcomes, statistical methods, and performance metrics. In addition, it emphasises the assessment of model performance and external validation to support synthesis across studies.
2. Refine the data extraction items: *TRIPOD* + *AI* and *TRIPOD-Cluster*
  The *Transparent Reporting of Multivariable Prediction Models for Individual Prognosis or Diagnosis* (*TRIPOD*) statement was published in 2015 to provide the minimum reporting recommendations for studies developing or evaluating the performance of a prediction model. It was updated to *TRIPOD* + *AI* [26] (Additional file 3) in 2024 to adapt to the developing prediction models applying artificial intelligence. *TRIPOD* + *AI* provides harmonised guidance for reporting prediction model studies, irrespective of whether regression modelling or machine learning methods have been used. As a process version between them, the *TRIPOD-Cluster* [27, 28] (Additional file 4) framework emphasises the necessity of considering cluster heterogeneity during the development or validation of models based on multi-cluster data.
3. Risk of bias assessment: *PROBAST* + *AI*
  The *Predictive Model Bias Risk Assessment Tool* + *AI* (*PROBAST* + *AI*, Additional file 5) [29], an updated version of *PROBAST* [30] released in 2025 to accommodate AI methodologies, is a specialised quality assessment guideline (or risk of bias tool) developed for studies on the development or validation of prediction models. Building upon a clear articulation of the research question, the tool identifies potential sources of bias across four key domains: Participants, Predictors, Outcome, and Model Development/Validation. This risk of bias is subsequently rated as ‘high’, ‘low’, or ‘unclear’. It also requires assessors to determine the model’s applicability.

**Fig. 1:**
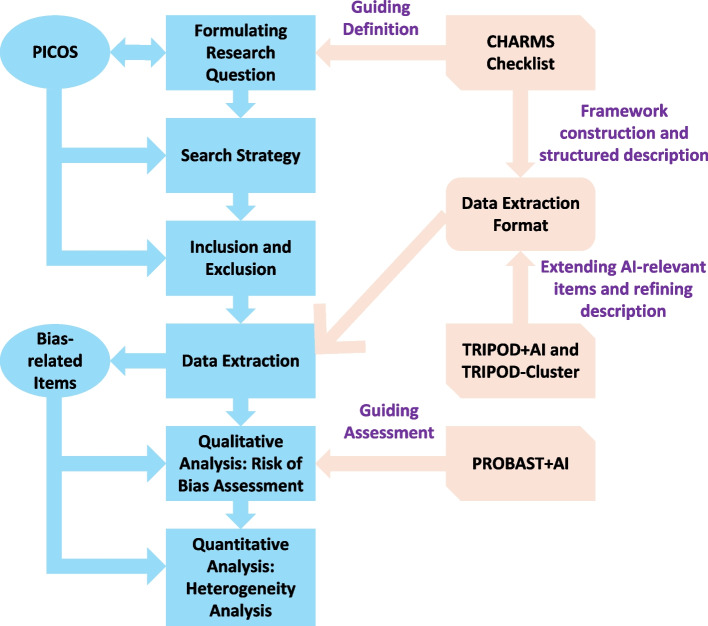
Structured workflow linking review stages to methodological guidelines

To clarify the relationships between various guidelines and outline the application process of different research tools, a workflow chart (Fig. 1) and Table 1 have been developed to visualise the integrated workflow.

**Table 1:** Methodological guidelines in the workflow

Guideline	Workflow
*CHARMS* checklist	Defines the review question (PICOS), specifies the data-extraction framework, and structures the narrative description (detailing what to extract and how it is organised)
*TRIPOD* + *AI*	Extends the extraction form by incorporating AI-relevant items (e.g. model output, imbalance sampling, hyperparameter) and transparency items (e.g. data/code sharing)
*TRIPOD-Cluster*	Refines the structured description to record dataset clustering and between-cluster performance, enabling heterogeneity profiling and flagging fairness concerns for subsequent appraisal
*PROBAST* + *AI*	Evaluates methodological quality (risk of bias and applicability) based on the information extracted. It assesses study validity, rather than model performance

### Search strategy

This review will implement a comprehensive search strategy following the *JBI* three-phase approach [31] with the help of a library informaticist. An initial limited search of *PubMed* and *Embase* was performed to roughly identify relevant articles. Search terms have been defined based on the keywords and index terms according to the participant, intervention, comparator, and outcome framework. This will be followed by a search using all identified combinations of subject terms (e.g. *MeSH* terms and *Emtree* terms) and free terms across six international databases (*PubMed*, *Embase*, *Scopus*, *Web of Science*, *IEEE Xplore*, and *ACM*) and four Chinese databases (*CNKI*, *WanFang*, *VIP*, and *SinoMed*). We will search for grey literature in *ProQuest Dissertations & Theses*, as well as in the dissertation databases of *CNKI* and *WanFang*, and we will also utilise *Google Scholar* to screen the reference lists of included studies for additional relevant records. Complete search strategies will be found in Additional file 6.

### Inclusion and exclusion criteria

Based on the *CHARMS* checklist and the pre-searched literature, the inclusion and exclusion criteria of the literature are considered from five perspectives: targeted population, types of index prediction models, types of comparator prediction models, types of predicted outcomes, and types of studies.

1. Targeted population: Maternal, newborns, and children under five
  - Inclusion criteria: Studies that concentrate on the population of maternal, newborns, and children under five.
  - Exclusion criteria: Studies focusing exclusively on disease-specific populations (e.g. sepsis, newborn pneumonia, HIV, eclampsia), as such models predict case fatality rather than overall mortality.
2. Types of index prediction models: AI-powered prediction models
  - Inclusion criteria: Studies that use AI-powered prediction models, specifically are defined as utilising advanced algorithms such as machine learning, decision tree, or neural network.
  - Exclusion criteria: Studies that use AI for data mining, extraction, or analysis only.
3. Types of comparator prediction models: Prediction models using traditional approaches
  - Inclusion criteria: Studies that use comparator prediction models with specific calculation bases (e.g. *Cox* regression, trend analysis) or traditional algorithms (e.g. logistic regression).
  - Exclusion criteria: Studies that use prediction methods with index scores only or lack specific calculation bases.
4. Outcomes to be predicted: The mortalities of maternal, newborns, and children under five, including but not limited to the following ratio indexes, and any related death and survival
  - Inclusion criteria: Studies that report mortality rates in the targeted population, including maternal mortality ratio (MMR), neonatal mortality rate (NMR), and under-five mortality rate (U5MR); studies that report death or survival numbers/ratios in the targeted population.
    According to the official calculation methods of the *WHO*, the definitions and calculation formulas of these outcome indicators are as follows:
    1. MMR: (Number of maternal deaths / Number of live births) × 100,000. $\mathrm{MMR}=\frac{\text{Number of maternal deaths}}{\text{Number of live births}}\times \mathrm{100,000}$
    2. NMR: (Number of neonatal deaths / Number of live births) × 1000. $\mathrm{NMR}=\frac{\text{Number of neonatal deaths}}{\text{Number of live births}}\times 1000$
    3. U5MR: (Number of deaths of children under five / Number of live births) × 1000. $\mathrm{U}5\mathrm{MR}=\frac{\text{Number of deaths of children under five}}{\text{Number of live births}}\times 1000$
  - Exclusion criteria: Studies that predict non-mortality outcomes (e.g. disease incidence) without mortality as the modelled endpoint. To specify, the above indices are solely reported as background, descriptive context, or predictors/covariates.
5. Types of studies: Development and/or evaluation studies of prognostic models
  - Inclusion criteria: Any research developing or evaluating prognostic models based on reasonable data sources, including cohort studies, case-control studies, randomised controlled trials, and existing databases.
  - Exclusion criteria: Studies that either cannot extract specific outcome measures or data, or only have abstracts available without full text access (e.g. conference abstract).

**Fig. 2:**
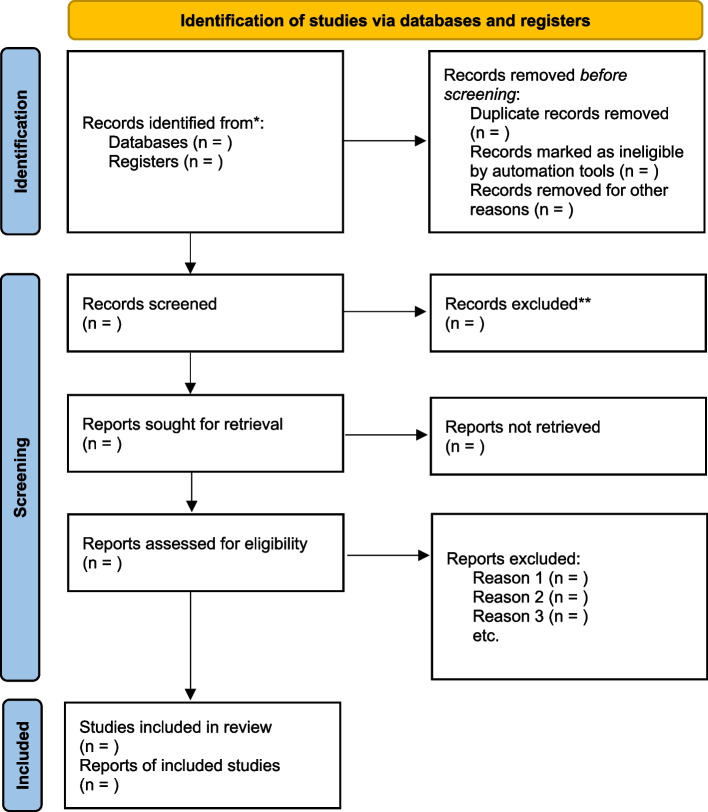
PRISMA 2020 flow diagram for new systematic reviews which included searches of databases and registers only. *Consider, if feasible to do so, reporting the number of records identified from each database or register searched (rather than the total number across all databases/registers). **If automation tools were used, indicate how many records were excluded by a human and how many were excluded by automation tools. Source: Page MJ, et al. BMJ 2021;372:n71. https://doi.org/10.1136/bmj.n71. This work is licensed under CC BY 4.0. To view a copy of this license, visit https://creativecommons.org/licenses/by/4.0/

Based on the above eligibility criteria, research team members will independently assess studies by screening the title, abstract, and full text in order to remove duplicates. *Endnote20* will be employed to ensure efficient and effective execution. In addition, we will endeavour to obtain full papers by contacting authors of papers not published in full online. Any disagreements will be resolved through collaborative discussion or by involving a third-party, more experienced researcher to reach a consensus. To summarise, a *PRISMA2020* flow diagram (Fig. 2) will document the study selection process.

### Data extraction

This review will be based on the above methodological guidelines (*CHARMS* checklist, *TRIPOD* + *AI* checklist, and *TRIPOD-Cluster* checklist) for data extraction. This protocol provisionally identifies the data extraction form (Table 2) applicable to this study, based on the entries of the above checklists and previous studies. Prior to formal data extraction, the form will be pre-tested and modified through group discussions based on the literature searched and the methodological guidelines mentioned above.

**Table 2:** Data extraction form (initial design) (explanation: the data extraction form entries were derived from CHARMS checklist, TRIPOD + AI, and TRIPOD-Cluster; and the sections were divided according to the PICOS principle)

Section	Criteria	Description
1. Study basic information (3 entries)	1.1 Author (year)* (D^a^; E^b, c^)	Authors of the article and year of publication
	1.2 Country (D; E)	Country/countries on which the study is based
	1.3 Objectives* (D; E)	Whether to develop or validation of a prediction model (or both)
2. Participants (8 entries)	2.1 Source of data* (D; E)	Description of the data source (e.g. cohort, case–control, randomised trial participants, or registry data), the rationale for using these data, and representativeness of the data
	2.2 Dates of collected data (D; E)	Specify the dates of the collected data, including start and end of participant accrual; and, if applicable, end of follow-up
	2.3 Settings* (D; E)	Specify key elements of study setting (e.g. primary care, secondary care, hospital, and general population) including the number and location of centres
	2.4 Eligibility criteria* (D; E)	Details on how participants were recruited and eligibility criteria
	2.5 Handling of missing data* (D; E)	Methods used to handle missing data. Whether reasons were provided for omitting any data
	2.6a Participant flow* (D; E)	The flow of clusters (if available) and participants through the study, including the number of participants with and without outcome and, if applicable, a summary of the follow-up time
	2.6b Sample size* (D; E)	The number of sample size, as well as explanations on how the study size was arrived at (separately for development and evaluation) and justification that the study size was sufficient to answer the research question, including details of any sample size calculation
	2.6c Participant description* (D; E)	Characteristics of the study clusters (if available) and participants and, if applicable, for each data source or setting, including the key dates, key predictors (including demographics), treatments received, sample size, number of outcome events, follow-up time, and amount of missing data
3. Index and comparator models (13 entries)	3.1a Predictor selection (initial)* (D)	The timing and method of predictor selection
	3.1b Predictor selection during modelling* (D)	Methods and criteria used for selecting predictors during model development
	3.2 Distribution of predictors* (D; E)	Details on the number and type of predictors used in the model, including how predictor weights were adjusted. Provided reasons if subjective adjustment required
	3.3 Blinding of predictor assessment* (D; E)	Any actions to blind assessment of predictors for the outcome and other predictors
	3.4 Definition of candidate predictors* (D; E)	Definitions of candidate predictors. If predictor measurement requires subjective interpretation, whether had descriptions on the qualifications and demographic characteristics of the predictor assessors
	3.5 Events per variable (D; E)	Ratio of events to the number of predictors
	3.6 Data preparation* (D)	Data pre-processing and quality checking (e.g. data cleaning), and whether this was similar across relevant sociodemographic groups
	3.7a Modelling method* (D)	How the data were used for development and evaluation of model performance, including whether the data were partitioned, considering any sample size requirements
	3.7b Handling of predictors in the modelling* (D)	How predictors were handled in the model (functional forms, rescaling, transformations, or any standardisation)
	3.7c Model description* (D; E)	Specify the type of model, rationale, all model-building steps, including any hyperparameter tuning, and methods for internal validation
	3.7d Algorithms* (D; E)	How the model predictions were calculated (e.g. formula, code, object, application programming interface)
	3.8 Imbalance model handling* (D; E)	If class imbalance methods were used, why and how this was done, and any subsequent methods to recalibrate the model or the model predictions
	3.9 Model assumption (E)	Whether the model’s assumptions were met
4. Study outcomes (16 entries)	4.1 Presentation of models* (D; E)	How the final and other models were presented
	4.2 Alternative presentations of the final prediction models (D; E)	Other ways the prediction model was presented
	4.3 Definition and method for measurement of outcome* (D)	Specify definition and method of the mortality/deaths/survival in study clusters (if available) and individuals
	4.4 Consistency of outcome measurement* (D)	Whether the same outcome (mortality, etc.) definition was used for all participants, and any missing data reported
	4.5 Type of outcome* (D)	Description of the outcome (mortality, etc.) type (e.g. single or combined endpoints)
	4.6 Relationship between candidate predictors and the outcome (D; E)	If predictors were part of the outcome assessment (e.g. in panel or consensus diagnosis)
	4.7 Blinding of outcome assessment* (D; E)	Whether and how the outcome was assessed without knowledge of the predictors
	4.8a Model output* (D)	Specify the output of the prediction model (e.g. probabilities, classification). Details and rationale for any classification and how the thresholds were identified
	4.8b Classification measurements* (D; E)	Performance metrics related to classification and whether a priori cut points were used
	4.8c Method used for testing model performance* (D; E)	Techniques or calculations used to evaluate model performance: development dataset only or separate external validation
	4.9 Model performance* (D; E)	Model performance estimates with confidence intervals, including for any key subgroups (e.g. sociodemographic, settings)
	4.10 Model performance evaluation* (D; E)	How all measures and plots were used (and their rationale) to evaluate model performance (e.g. discrimination, calibration, clinical utility) and, if relevant, to compare multiple models
	4.11a Heterogeneity* (D; E)	Comparison with the development data of the distribution of important variables (demographics, predictors, and outcome)
	4.11b Heterogeneity handling* (D; E)	If and how any heterogeneity in estimates of model parameter values and model performance was handled and quantified across clusters (e.g. settings, countries)
	4.11c Subsequent actions towards heterogeneity (D; E)	The results of any across-cluster heterogeneity assessments that led to subsequent actions during developing (e.g. inclusion or exclusion of predictors or clusters)
	4.12 Model updating (E)	Results from any model updating, including the updated model and subsequent performance
5. Study usability (3 entries)	5.1a Data sharing* (D; E)	Details of the availability of the study data
	5.1b Code sharing* (D; E)	Details of the availability of the analytical code and of the full prediction model, especially to allow predictions in new individuals and to enable third-party evaluation and implementation, including any restrictions to access or re-use (e.g. freely available, proprietary)
	5.2 Quality assessment* (D; E)	For model developing studies, specify how poor quality or unavailable input data (e.g. predictor values) should be assessed and handled when implementing the prediction model. For model evaluating studies, specify the quality assessment of the models and research

^*^Key entries for follow-up qualitative, quantitative, and risk of bias assessment

^a^D = items relevant only to the development of a prediction model

^b^E = items relating solely to the evaluation of a prediction model

^c^D; E = items applicable to both the development and evaluation of a prediction mode

The formal data extraction process will be carried out independently by a research team, with each member responsible for a different section to ensure accuracy and reproducibility. Discrepancies in data extraction results will be resolved through collaborative discussion and may require guidance from another senior researcher if necessary. If data is incomplete, researchers will note this carefully and attempt to contact the original authors of the study to obtain missing information or additional clarification if necessary. The researcher will systematically organise the data collected using *Excel* spreadsheets or specialist software applications to ensure efficient handling of the data and ease of access throughout the review process.

### Qualitative analysis

Qualitative analyses will include quality assessment, heterogeneity assessment, and risk of bias assessment. Study members will be divided into two groups to perform the analyses back-to-back, and any conflicts will be referred to a more specialised and experienced third member for discussion and adjudication.

#### Quality assessment

The quality of each study will be assessed based on the data extraction form. We will evaluate (i) completeness and transparency of reporting, focusing on items that may introduce bias and noting any not-informed entries, and identify key items overlooked in prior work; and (ii) the appropriateness of modelling workflows for development and validation (e.g. potential data leakage), which informs the analysis of methodological heterogeneity and risk of bias. Findings will be summarised in overview tables and charts highlighting reporting gaps, workflow concerns, and study-level strengths.

#### Heterogeneity assessment

Heterogeneity assessment will include not only clustering heterogeneity indicated by multicentre data sources, but also the methodological heterogeneity of subsequent integration of studies (statistical heterogeneity will be analysed in the subsequent quantitative synthesis, if performed). The focus of this section is to identify heterogeneity both within and between studies. Heterogeneity will be assessed by characteristics of populations, study settings, distribution of predictors, validation methods, etc. It should be noted that internal heterogeneity within a study’s population will be flagged as a possible indicator of fairness bias. The above elements are included in the data extraction form. The results of the heterogeneity analyses of the clusters will be presented in descriptive tables or figures.

#### Risk of bias assessment

The risk of bias of the included studies will be assessed using *PROBAST* + *AI*. This review will assess the risk of bias for each study in four main areas: study population, predictors, outcome indicators, and data analyses, and will ultimately define the risk of bias based on the question and type of study as ‘high’, ‘low’, or ‘unclear’. In addition, the applicability of the study in the first three domains will also be assessed. Conflicting results of bias assessment, if any, will be resolved through discussion, and senior researchers will be consulted if necessary. The results of the risk of bias assessment will be presented in tabular or graphical form.

### Quantitative analysis

The quantitative analysis process will be divided into two main parts. First, we will integrate the models used in the included studies, the predictors and performance of the models, and make a rough comparison of the performance of AI and traditional models in predicting maternal, neonatal, and under-five mortality. In addition, if data permit, we will complete a meta-analysis compiling results from multiple studies, thereby measuring overall effect sizes and assessing the consistency of findings.

#### Data synthesis and analysis

Algorithms and predictors of the models.

The modelling algorithms included in the study and the predictors they use will be statistically integrated in this section. The number of occurrences of model algorithms and predictors will be presented in tables or charts (with the population characteristics and settings they are applied to).

Model performance.

The performance of the included studies will be counted or calculated (if data are sufficient). In our context, performance is summarised in three domains: discrimination, calibration, and classification measures:

1. Discrimination: The model’s ability to separate individuals who will die from those who will survive (i.e. high- vs low-risk). A common metric is the C-statistics
  C-statistics: C-statistics, also known as the area under the receiver operating characteristic curve (AUROC), refers to the line connecting various points drawn under specific stimulus conditions, with sensitivity (see below) as the horizontal axis and false positive rate (1 − specificity, see below) as the vertical axis. The larger the AUC value, the better the classification performance of the model.
2. Calibration: The agreement between predicted and observed risks. Typical summaries include O/E ratio, calibration plots, calibration slope, and tests such as the Hosmer–Lemeshow test
  O/E ratio: O/E ratio refers to the ratio of the actual number of observed events (O) and the predicted number of events that will occur (E). It is used to evaluate the calibration of the model.
3. Classification measures: Threshold-based measures derived from the confusion matrix (TP, true positive; TN, true negative; FP, false positive; FN, false negative)
  Accuracy: Accuracy refers to the proportion of correctly classified samples to the total number of samples. $\mathrm{Accuracy}=\frac{\mathrm{TP}+\mathrm{TN}}{\mathrm{TP}+\mathrm{TN}+\mathrm{FP}+\mathrm{FN}}$
  Sensitivity: Sensitivity, also called recall, refers to the proportion of correctly classified positive samples to the number of samples determined by the classifier as positive samples. $\mathrm{Sensitivity}=\frac{\mathrm{TP}}{\mathrm{TP}+\mathrm{FN}}$
  Specificity: Specificity refers to the proportion of correctly classified negative samples to the number of samples determined by the classifier as negative samples. $\mathrm{Specificity}=\frac{\mathrm{TN}}{\mathrm{TN}+\mathrm{FP}}$
  Positive predictive value (PPV): PPV, also called precision, refers to the proportion of correctly classified positive samples (TP) among all samples predicted as positive. $\mathrm{PPV}=\frac{\mathrm{TP}}{\mathrm{TP}+\mathrm{FP}}$
  Negative predictive value (NPV): NPV refers to the proportion of correctly classified negative samples (TN) among all samples predicted as negative. $\mathrm{NPV}=\frac{\mathrm{TN}}{\mathrm{TN}+\mathrm{FN}}$

In this section, a rough comparison will be made between the performance of AI-powered prediction models and traditional prediction models based on different populations and settings, as well as the performance of AI-powered prediction models applying different algorithms. The results will be presented in tables or figures.

#### Meta-analysis

##### Meta-analysis incorporated into qualification

In previous reviews of this type, the criterion for inclusion of models in meta-analyses has often been ≥ 3 external validations. However, due to the rapid development of models relevant in this field and the delayed nature of external validation, only a fairly small number of models can match the criteria for external validation. This review will adjust the inclusion criteria for studies entering the meta-analysis based on the results of the qualitative analyses described above, as follows:

1. Studies with ≥ 3 external validations.
2. Studies that underwent ≥ 2 external validations and were determined to be of high quality, low cohort heterogeneity (both within and between studies), and low/unknown bias.
3. If the number of studies available for inclusion is sufficient, this study will undergo subsequent meta-analysis for more structured results.

##### Meta-analysis

Meta-analyses will be performed in different populations (maternal, neonatal, and under-five children). During this process, dichotomous variables, like the O/E ratio, will be estimated with the corresponding 95% CI. For continuous variables, like C-statistics, we will calculate pooled effect sizes by mean difference (MD) with 95% CI.

Cochran’s *Q* and *I*^2^ tests will be used to quantify statistical heterogeneity and the selective effects models. Typically, such a review will employ a random-effects model as it provides more conservative estimates by accounting for variations between studies, offering a more robust and generalisable result.

In this review, sensitivity analyses will be conducted by continuously excluding one study at a time to confirm the reliability and stability of meta-analysis results. Besides, depending on recent guidelines, such as those discussed in *PROBAST*, the evaluation of publication bias for effect models is not heavily emphasised. Therefore, we will not assess publication bias for this model.

Finally, comprehensive subgroup analyses will be based on different settings, which aim to summarise the key operational aspects of AI-powered models in predicting mortality in maternal, newborn, and children under five. All the analyses will be completed by *Stata* 18.0, and a *P* value of less than 0.05 will be considered statistically significant.

After completing the qualitative and quantitative analyses, the entire process will also be assessed according to the Grading of Recommendations Assessment, Development and Evaluation (GRADE) [32] tool to determine the strength of evidence for this review.

## Discussion

This protocol outlines the methodology used in a systematic review of artificial intelligence prediction models for predicting maternal, neonatal, and under-five mortality. This review has two objectives. The first is to assess the quality of the original research through the more established guidelines currently available. The second is to integrate current research comparing the performance of AI-powered prediction models with traditional prediction models.

However, both of these objectives are currently methodologically immature. We anticipate several key methodological challenges in this review:

- First, comprehensively identifying potential sources of bias and heterogeneity. Key design details, such as data source, predictor measurement, and heterogeneity across clusters, are often omitted. To address this, our approach involves employing a structured taxonomy for bias and heterogeneity sources. When essential information is missing, we will apply targeted assumptions based on explicit rationale.
- Second, setting principled and transparent inclusion standards for quantitative synthesis is nontrivial. Current meta-analysis eligibility thresholds in this protocol remain largely experience-based and aspirational; consequently, overly strict criteria risk arbitrarily excluding the majority of available studies. We will therefore pre-define tiered synthesis rules: (a) the primary meta-analysis will prioritise studies reporting externally validated results; (b) a secondary synthesis will permit high-quality development studies with only internal validation when external validation is unavailable; and (c) a narrative synthesis will be used when key metrics are not harmonisable. Each tier of synthesis will be reported with full transparency.
- Finally, choosing appropriate synthesis models must account for statistical dependencies. We anticipate that most included studies will contribute two or more models, which introduces within-study dependence. To account for this, we will apply robust variance estimation (RVE), which incorporates intra-study correlations to adjust the variance. Furthermore, it is crucial to recognise that sensitivity and specificity are inherently negatively correlated. Therefore, the application of bivariate models (such as the hierarchical summary ROC model) is the appropriate solution to jointly synthesise these metrics.

This systematic review of high-quality studies will provide evidence in support of or against the hypothesis that AI-powered models provide more accurate and sensitive predictions compared with traditional models. We will adhere strictly to the recommended methodology for systematic review, data extraction, and risk of bias assessment, with results reported in accordance with the *PRISMA2020* guidelines. Additionally, if the data permits, a meta-analysis will be conducted to compile results from multiple studies, measuring overall effect sizes and evaluating the consistency of findings. This review is expected to fill gaps in the field and outline the methods for evaluating, synthesising, and comparing existing studies, providing insights that could significantly contribute to the advancement of AI in maternal and child healthcare.

## Supplementary Information

Additional file 1. PRISMA-P 2015 checklist.

Additional file 2. CHARMS 2014 relevant items to extract from individual studies in a systematic review of prediction models.

Additional file 3. TRIPOD+AI checklist.

Additional file 4. TRIPOD-Cluster checklist of items to include when reporting a study developing or validating a multivariable prediction model using clustered data.

Additional file 5. PROBAST+AI tool.

Additional file 6. Specific search strategies and results of each database.

## Data Availability

Not applicable.

## Abbreviations

AI: Artificial intelligence
CHARMS: Checklist for Critical Appraisal and Data Extraction for Systematic Reviews of Prediction Modelling Studies
CI: Confidence interval
GDP: Gross domestic product
GRADE: Grading of Recommendations Assessment, Development and Evaluation
MMR: Maternal mortality ratio
NMR: Neonatal mortality rate
O/E: Observed/expected ratio
PPV: Positive predictive value
NPV: Negative predictive value
PROBAST: Prediction Model Risk of Bias Assessment Tool
PROBAST + AI: Prediction Model Risk of Bias Assessment Tool + Artificial Intelligence
PRISMA: Preferred Reporting Items for Systematic Reviews and Meta-Analyses
PRISMA-P: Preferred Reporting Items for Systematic Reviews and Meta-Analyses Protocols
ROC: Receiver operating characteristic
SDGs: Sustainable Development Goals
SMD: Standardised mean difference
TRIPOD: Transparent Reporting of a Multivariable Prediction Model for Individual Prognosis or Diagnosis
TRIPOD + AI: Transparent Reporting of a Multivariable Prediction Model for Individual Prognosis or Diagnosis + Artificial Intelligence
TRIPOD-SRMA: Transparent Reporting of a Multivariable Prediction Model for Individual Prognosis or Diagnosis—Systematic Reviews and Meta-Analyses
