# Supplementary material for: Artificial intelligence-powered models in predicting mortality in maternal, newborn, and children under five: a systematic review protocol
Source: Syst Rev. 2026 Mar 7;15:134. doi: 10.1186/s13643-026-03138-5 (PMC13081567; doi:10.1186/s13643-026-03138-5)
Supplement: Supplementary file 4 — Additional file 4. TRIPOD-Cluster checklist of items to include when reporting a study developing or validating a multivariable prediction model using clustered data. [file 13643_2026_3138_MOESM4_ESM.pdf]

TRIPOD-Cluster checklist of items to include when reporting a study developing or validating a multivariable prediction model using clustered data

#	Description	Page #
**Title and abstract**		
1	Identify the study as developing and/or validating a multivariable prediction model, the target population, and the outcome to be predicted.	
2	Provide a summary of research objectives, setting, participants, data source, sample size, predictors, outcome, statistical analysis, results, and conclusions.*	
**Introduction**		
3a	Explain the medical context (including whether diagnostic or prognostic) and rationale for developing or validating the prediction model, including references to existing models, and the advantages of the study design.*	
3b	Specify the objectives, including whether the study describes the development or validation of the model.*	
**Methods**		
4a	Describe eligibility criteria for participants and datasets.*	
4b	Describe the origin of the data, and how the data were identified, requested, and collected.	
5	Explain how the sample size was arrived at.*	
6a	Define the outcome that is predicted by the model, including how and when assessed.*	
6b	Define all predictors used in developing or validating the model, including how and when measured.*	
7a	Describe how the data were prepared for analysis, including any cleaning, harmonisation, linkage, and quality checks.	

#	Description	Page #
7b	Describe the method for assessing risk of bias and applicability in the individual clusters (eg, using PROBAST).	
7c	For validation, identify any differences in definition and measurement from the development data (eg, setting, eligibility criteria, outcome, predictors).*	
7d	Describe how missing data were handled.*	
8a	Describe how predictors were handled in the analyses.	
8b	Specify the type of model, all model-building procedures (eg, any predictor selection and penalisation), and method for validation.*	
8c	Describe how any heterogeneity across clusters (eg, studies or settings) in model parameter values was handled.	
8d	For validation, describe how the predictions were calculated.	
8e	Specify all measures used to assess model performance (eg, calibration, discrimination, and decision curve analysis) and, if relevant, to compare multiple models.	
8f	Describe how any heterogeneity across clusters (eg, studies or settings) in model performance was handled and quantified.	
8g	Describe any model updating (eg, recalibration) arising from the validation, either overall or for particular populations or settings.*	
9	Describe any planned subgroup or sensitivity analysis, (eg, assessing performance according to sources of bias, participant characteristics, setting).	
**Results**		

#	Description	Page #
10a	Describe the number of clusters and participants from data identified through to data analysed. A flow chart may be helpful.*	
10b	Report the characteristics overall and where applicable for each data source or setting, including the key dates, predictors, treatments received, sample size, number of outcome events, follow-up time, and amount of missing data.*	
10c	For validation, show a comparison with the development data of the distribution of important variables (demographics, predictors, and outcome).	
11	Report the results of the risk of bias assessment in the individual clusters.	
12a	Report the results of any across-cluster heterogeneity assessments that led to subsequent actions during the model's development (eg, inclusion or exclusion of particular predictors or clusters).	
12b	Present the final prediction model (ie, all regression coefficients, and model intercept or baseline estimate of the outcome at a given time point) and explain how to use it for predictions in new individuals.*	
13a	Report performance measures (with uncertainty intervals) for the prediction model, overall and for each cluster.	
13b	Report results of any heterogeneity across clusters in model performance.	
14	Report the results from any model updating (including the updated model equation and subsequent performance), overall and for each cluster.*	
15	Report results from any subgroup or sensitivity analysis.	
**Discussion**		
16a	Give an overall interpretation of the main results, including heterogeneity across clusters in model	

#	Description	Page #
	performance, in the context of the objectives and previous studies.*	
16b	For validation, discuss the results with reference to the model performance in the development data, and in any previous validations.	
16c	Discuss the strengths of the study and any limitations (eg, missing or incomplete data, non-representativeness, data harmonisation problems).*	
17	Discuss the potential use of the model and implications for future research, with specific view to generalisability and applicability of the model across different settings or (sub)populations.*	
**Other information**		
18	Provide information about the availability of supplementary resources (eg, study protocol, analysis code, datasets).*	
19	Give the source of funding and the role of the funders for the present study.	

A separate version of this checklist is available in the supplementary table.

PROBAST=prediction model risk-of-bias assessment tool.

- * Item text is an adaptation of one or more existing items from the original TRIPOD (transparent reporting of a multivariable prediction model for individual prognosis or diagnosis) checklist.
