# Supplementary material for: Artificial intelligence-powered models in predicting mortality in maternal, newborn, and children under five: a systematic review protocol
Source: Syst Rev. 2026 Mar 7;15:134. doi: 10.1186/s13643-026-03138-5 (PMC13081567; doi:10.1186/s13643-026-03138-5)
Supplement: Supplementary file 5 — Additional file 5. PROBAST+AI tool. [file 13643_2026_3138_MOESM5_ESM.pdf]

Supplementary Table 5. The subsequent steps for use of the PROBAST+AI tool ,conform the use of the original PROBAST tool.

Cite as: Moons KGM, Damen JAA, Kaul T, et al. PROBAST+AI: an updated quality, risk of bias, and applicability assessment tool for prediction models using regression or artificial intelligence methods. *BMJ* 2025;388:e082505 doi:10.1136/bmj-2024-082505

Step	Task	When to complete
1	Specify the intended purpose of the prediction model assessment or of the prediction model systematic review	Once per assessment or systematic review
2	Classify the type of prediction model study (development or evaluation or both)	Once for each prediction model of interest in each publication assessed, for each relevant outcome
3	Assess quality and applicability to the intended purpose of the developed prediction model, for each domain<br>&<br>Assess risk of bias and applicability to the intended purpose of the evaluated prediction model, for each domain	Once for each model development for each distinct prediction model in a publication<br><br>Once for each model evaluation for each distinct prediction model in a publication
4	Assess the overall quality and applicability for model development, and separately, the risk of bias and applicability for model evaluation	Once for each assessed prediction model in a publication, and separately for model development and for model evaluation

Directly based on PROBAST-2019 (www.probast.org). We strongly recommend reading the Explanation and Elaboration Light (Supplementary Table 4) and the Explanation and Elaboration document of PROBAST-2019[1]. We also recommend checking the example papers on www.probast.org.

[1] Moons KGM, Wolff RF, Riley RD, Whiting PF, Westwood M, Collins GS, et al. PROBAST: A Tool to Assess Risk of Bias and Applicability of Prediction Model Studies: Explanation and Elaboration. Ann Intern Med. 2019;170(1):W1-w33

Step 1: Use the PICOTS guidance to specify your intended purpose or aim of the prediction model assessment, and the review question in case a systematic review of prediction model studies is conducted

PICOTS:

Item	Explanation
**Population**	Define the target population (e.g., patients) in whom the assessed prediction models are to be applied. The target population not only directs search strings and in/exclusion criteria of prediction models or prediction model studies in case of a systematic literature review, but also directs the applicability assessment.
**Index model(s)**	Define the targeted prediction models to be assessed, which may be a single prediction model (the index model) of which the predictive accuracy is meta-analysed across multiple external evaluation studies of that index model but may also address multiple prediction models (developed or evaluated) for the targeted population, outcome or setting, depending on the assessor's or prediction model review focus.
**Comparator model(s)**	Define the other prediction models whose predictive ability is compared to that of the index model.
**Outcome(s)**	Define the outcomes or endpoints that are predicted by the index (and possibly comparator) prediction models in the target population.
**Timing**	1. Define the moment or time-point (e.g., in the patient work-up) at which the prediction with the prediction models is made (i.e., the start point or T0 of the use of the models).<br>2. Define the time or follow-up period in which the outcomes are being predicted by the prediction models in the targeted population (prediction horizon).
**Setting and intended use of the prediction model**	Define the healthcare setting or context to which the index prediction models apply. The prediction ability of models may change across healthcare settings or contexts.

The following table should be completed once per prediction model review.

**Population**	
**Index model(s)**	
**Comparator model(s)**	
**Outcome(s)**	
**Timing**	
**Setting and intended use of the prediction model**	

Step 2: Classify the type of prediction model assessment

Use the following table to classify the assessments as model development, model evaluation, or combination (see also Box 1 in the paper). Different signalling questions apply for different types of prediction model assessments. If the assessment does not fit one of these classifications, then PROBAST+AI should not be used.

**Classify the assessment based on its aim**			
Type of prediction study	*Explanation*	*PROBAST+AI boxes to complete*	*Tick as appropriate*
Development only	Prediction model development only, i.e., without evaluation of its performance.	Model development	
Evaluation only	External validation of one or more existing models in new data	Model evaluation	
Combination	Prediction model development combined in the same study (publication) with the evaluation of its apparent performance, internal validation performance, or external validation performance.	Model development and evaluation	

Step 3: Assess quality and applicability or risk of bias and applicability

This table should be completed once for each publication being assessed and for each relevant outcome in the prediction model review

**Publication reference**	
**Models of interest**	
**Outcome of interest**	

PROBAST+AI: MODEL DEVELOPMENT

Each domain of the model development part is judged for concerns about quality (low, high, or unclear concern). Each domain includes signalling questions to help make judgements. Signalling questions are rated as yes (Y), probably yes (PY), probably no (PN), no (N), no information (NI), and in some cases not applicable (NA, items marked with an asterisk*). All signalling questions are phrased so that 'yes' or 'probably yes' indicate low concerns for quality (= high quality).

Any signalling question rated as 'no' or 'probably no' flags the potential for sincere concerns regarding quality of that domain. You will need to use your judgement to determine whether the entire domain should be rated as 'high', 'low', or 'unclear' concern regarding quality. The Explanation & Elaboration Light (Supplementary Table 4) contains further information and examples on rating signalling questions and concerns regarding quality for each domain of the model development process.

The first three domains are also rated for concerns regarding applicability (low/high/unclear) of the prediction model (study) to the review question or to intended use of the assessed prediction models (as defined in step 1).

**DOMAIN 1: Participants and data sources**	
**A. Quality**	
Describe the sources of data and criteria for participant selection:	
	**Y/ PY/ PN/ N/ NI**
**1.1 Were appropriate data sources used?**	
**1.2 Was an appropriate study design used?**	
**1.3 Did the in- and exclusions of study participants result in a representative dataset?**	
**Concern regarding quality of selection of participants and data sources**	**QUALITYCONCERN:** ***low/high/unclear***
Rationale of quality rating:	
**B. Applicability**	
Describe included data sources, participants, setting, and dates:	
**Concern that the (data of the) included participants do not match the review question or the assessor's intended use of the prediction model**	**APPLICABILITY CONCERN:** ***low/high/unclear***

Rationale of applicability rating:

DOMAIN 2: Predictors	
**A. Quality**	
List and describe predictors included in the final prediction model, how they were defined and assessed, and their timing of assessment:	
	**Y/ PY/ PN/ N/ NI**
**2.1 Were predictors defined and assessed in a similar way for all participants?**	
**2.2 Was any pre-processing of predictors similar for all participants?**	
**2.3 Were predictor assessments made without knowledge of outcome data?**	
**2.4 Were the predictors included in the model available at the time the model was intended to be used?**	
**Concern regarding the quality of the predictors or their assessment**	**QUALITY CONCERN:** ***low/high/unclear***
Rationale of quality rating:	
**Applicability**	
**Concern that the definition, pre-processing, assessment, or timing of assessment of the predictors in the model do not match the review question or the assessor's intended use**	**APPLICABILITY CONCERN:** ***low/high/unclear***
Rationale of applicability rating:	

**DOMAIN 3: Outcome**	
**A. Quality**	
Describe the outcome, how it was defined and determined, and the time interval between predictor assessment and outcome determination:	
	**Y/ PY/ PN/ N/ NI**
**3.1 Were outcomes defined and assessed appropriately?**	
**3.2 Were outcomes defined and assessed in a similar way for all participants?**	
**3.3 Were outcome assessments made without use or knowledge of predictor data?**	
**3.4 Was the time interval between predictor assessment and outcome assessment appropriate?**	
**Concern regarding quality of the outcome or its determination**	**QUALITY CONCERN:** ***low/high/unclear***
Rationale of quality rating:	
**B. Applicability**	
At what time point was the outcome determined:<br><br>*If a composite outcome was used, describe the relative frequency/distribution of each contributing outcome:*	
**Concern that the outcome, its definition, assessment, or timing of assessment do not match the review question or the assessor's intended use**	**APPLICABILITY CONCERN:** ***low/high/unclear***
Rationale of applicability rating:	

**DOMAIN 4: Analysis**	
**Quality**	
Describe the numbers of participants, number of candidate predictors, number of outcome events:	
Describe how the prediction model was developed (e.g., with respect to modelling technique, predictor selection, and classification or risk group definition):	
Describe the performance measures of the prediction model, e.g., (re)calibration, discrimination, (re)classification, net benefit, and whether they were adjusted for optimism:	
Describe missing data on predictors and outcomes as well as methods used for handling these missing data:	
	**Y/ PY/ PN/ N/ NI/ NA**
**4.1 Was there evidence that the sample size was reasonable?**	
**4.2 Were continuous and categorical predictors handled appropriately?**	
**4.3 Were participants with missing or censored data handled appropriately in the analysis?**	
**4.4 If methods to address class imbalance were used, was the model or the model predictions recalibrated?***	
**4.5 Were methods used to address potential model overfitting?**	
**Concern regarding quality of the analysis**	**QUALITY CONCERN:** ***low/high/unclear***
Rationale of quality rating:	

PROBAST+AI: MODEL EVALUATION

Each domain of the model evaluation part is judged for risk of bias (low, high, or unclear). Each domain includes signalling questions to help make judgements. Signalling questions are again rated as yes (Y), probably yes (PY), probably no (PN), no (N), no information (NI), and in some cases not applicable (NA, items marked with an asterisk*). All signalling questions are phrased so that ‘yes’ or ‘probably yes’ indicate low risk of bias. Any signalling questions rated as ‘no’ or ‘probably no’ flags the potential for high risk of bias in that domain. You will need to use your judgement to determine whether the entire domain should be rated as ‘low’, ‘high’, or ‘unclear’ risk of bias.

If investigators only performed an apparent performance evaluation of the model, the responses to the signalling questions of domain 1, 2, and 3 in the Model Development section, can directly be copied and pasted to domain 1, 2, and 3 of this Model Evaluation section. However, the risk of bias judgement in the estimated model performance measures (e.g., their calibration or discrimination) still needs to be made.

Domain 4 should be evaluated separately for each type of model evaluation/validation assessed (apparent performance, internal validation, external validation). Shaded boxes indicate where signalling questions do not apply and should not be answered. The Explanation & Elaboration Light (Supplementary Table 4) contains further information and examples on rating signalling questions and risk of bias for each domain of model evaluation.

The first three domains are also rated for concerns regarding applicability (low/high/unclear) of the prediction model (study) to the review question or the intended use of the assessed prediction model(s) (as defined in step 1).

**DOMAIN 1: Participants and data sources**	
**A. Risk of bias**	
Describe the sources of data and criteria for participant selection:	
	**Y/ PY/ PN/ N/ NI**
**1.1 Were appropriate data sources used?**	
**1.2 Was an appropriate study design used?**	
**1.3 Did the in- and exclusions of study participants result in a representative dataset?**	
**Risk of bias introduced by the selection of participants and data sources**	**RISK OF BIAS:** ***low/high/unclear***
Rationale of risk of bias rating:	
**B. Applicability**	
Describe included data sources, participants, setting, and dates:	
**Concern that the (data of the) included participants do not match the review question or the assessor's intended use of the prediction model**	**APPLICABILITY CONCERN:** ***low/high/unclear***
Rationale of applicability rating:	

DOMAIN 2: Predictors	
**A. Risk of bias**	
List and describe predictors included in the evaluated model, e.g., definition and timing of assessment:	
	**Y/ PY/ PN/ N/ NI**
**2.1 Were predictors defined and assessed in a similar way for all participants?**	
**2.2 Was any pre-processing of predictors similar for all participants?**	
**2.3 Were predictor assessments made without knowledge of outcome data?**	
**2.4 Were the predictors included in the model available at the time the model was intended to be used?**	
**Risk of bias introduced by the predictors or their assessment**	**RISK OF BIAS:** ***low/high/unclear***
Rationale of risk of bias rating:	
**B. Applicability**	
**Concern that the definition, pre-processing, assessment, or timing of assessment of the predictors in the model do not match the review question or the assessor's intended use**	**APPLICABILITY CONCERN:** ***low/high/unclear***
Rationale of applicability rating:	

**DOMAIN 3: Outcome**	
**A. Risk of bias**	
Describe the outcome, how it was defined and determined, and the time interval between predictor assessment and outcome determination:	
	**Y/ PY/ PN/ N/ NI**
**3.1 Were outcomes defined and assessed appropriately?**	
**3.2 Were outcomes defined and assessed in a similar way for all participants?**	
**3.3 Were outcome assessments made without use or knowledge of predictor data?**	
**3.4 Was the time interval between predictor assessment and outcome assessment appropriate?**	
**Risk of bias introduced by the outcome or its determination**	**RISK OF BIAS:** ***low/high/unclear***
Rationale of risk of bias rating:	
**B. Applicability**	
At what time point was the outcome determined:<br><br>*If a composite outcome was used, describe the relative frequency/distribution of each contributing outcome:*	
**Concern that the outcome, its definition, assessment, or timing of assessment do not match the review question or the assessor's intended use**	**APPLICABILITY CONCERN:** ***low/high/unclear***
Rationale of applicability rating:	

DOMAIN 4: Analysis			
**Risk of bias**			
Describe numbers of participants, number of predictors, outcome events and events per predictor:			
Describe the performance measures of the evaluated model, e.g., (re)calibration, discrimination, (re)classification, net benefit, and whether they were adjusted for optimism:			
Describe any participants who were excluded from the analysis:			
Describe missing data on predictors and outcomes as well as methods used for handling these missing data:			
	**Y/ PY/ PN/ N/ NI/ NA**		
**4.1 Was model evaluation based on only apparent performance avoided?**			
	**A**	**I**	**E**
**4.2 Was there evidence that the sample size was reasonable?**			
**4.3 Were participants with missing or censored data handled appropriately in the analysis?**			
**4.4 If methods to address class imbalance were used, was the evaluation done in a dataset without imbalance correction?***			
**4.5 If data splitting was done to create training and test datasets, was there evidence that data leakage was avoided?***	**NA**		**NA**
**4.6 If resampling methods were used to evaluate model performance, were all model development steps replicated in the resampling process?***	**NA**		**NA**
**4.7 Was the predictive performance of the model evaluated appropriately, e.g., calibration, discrimination, and net benefit?**			
**Risk of bias introduced by the analysis**	**RISK OF BIAS:** ***low/high/unclear***		
Rationale of risk of bias rating:			

Step 4: Assess the overall concerns regarding quality, risk of bias and applicability of the prediction model

Use the following tables to reach overall judgements about concerns regarding quality and applicability for the model development process, and separately about risk of bias and concerns regarding applicability for the model performance evaluation. Complete for each assessed model.

Model development

OVERALL CONCERN REGARDING QUALITY OF THE PREDICTION MODEL DEVELOPMENT	
**Low concern regarding quality**	If all four domains were rated low concern regarding quality.
**High concern regarding quality**	If at least one domain was rated high concern regarding quality .
**Unclear concern regarding quality**	If at least one domain was rated unclear concern regarding quality and no domains were rated high concern.

OVERALL CONCERN FOR APPLICABILITY OF THE PREDICTION MODEL DEVELOPMENT	
**Low concern for applicability**	If all three domains were rated low concern for applicability.
**High concern for applicability**	If at least one domain was rated high concern for applicability.
**Unclear concern for applicability**	If at least one domain was rated unclear concern for applicability and no domains were rated high concern.

Model evaluation

OVERALL RISK OF BIAS OF THE PREDICTION MODEL EVALUATION	
**Low risk of bias**	If all four domains were rated low risk of bias.
**High risk of bias**	If at least one domain was rated high risk of bias.
**Unclear risk of bias**	If at least one domain was rated unclear risk of bias and no domains were rated high risk of bias.

OVERALL CONCERN FOR APPLICABILITY OF THE PREDICTION MODEL EVALUATION	
**Low concern for applicability**	If all three domains were rated low concern for applicability.
**High concern for applicability**	If at least one domain was rated high concern for applicability.
**Unclear concern for applicability**	If at least one domain was rated unclear concern for applicability and no domains were rated high concern.

OVERALL JUDGEMENT OF PREDICTION MODEL	
**Overall judgement of quality (development)**	**QUALITY CONCERN:** *low/high/unclear*
Summary of quality concern:	
**Overall judgement of risk of bias (evaluation)**	**RISK OF BIAS:** *low/high/unclear*
Summary of sources of potential bias:	
**Overall judgement of applicability (development)**	**APPLICABILITY CONCERN:** *low/high/unclear*
Summary of applicability concern:	
**Overall judgement of applicability (evaluation)**	**APPLICABILITY CONCERN:** *low/high/unclear*
Summary of applicability concern:	
