# Supplementary material for: Artificial intelligence-powered models in predicting mortality in maternal, newborn, and children under five: a systematic review protocol
Source: Syst Rev. 2026 Mar 7;15:134. doi: 10.1186/s13643-026-03138-5 (PMC13081567; doi:10.1186/s13643-026-03138-5)
Supplement: Supplementary file 6 — Additional file 6. Specific search strategies and results of each database. [file 13643_2026_3138_MOESM6_ESM.docx]

**Specific Search Strategies and Results of Each Database**

**Search Date: From Inception to** **11^st^ October 2024**

| **Database** | **Search Strategies** | **Results** |
| --- | --- | --- |
| PubMed | #1 "artificial intelligence"[MeSH Terms] OR "logistic models"[MeSH Terms] OR "support vector machine"[MeSH Terms] OR "neural networks, computer"[MeSH Terms] OR "decision trees"[MeSH Terms] OR "random forest"[MeSH Terms] OR "bayes theorem"[MeSH Terms] | 422,317 |
|  | #2 "artificial intelligence*"[Title/Abstract] OR "computational intelligence*"[Title/Abstract] OR "machine intelligence*"[Title/Abstract] OR "computer reasoning*"[Title/Abstract] OR "automated reasoning*"[Title/Abstract] OR "computer vision system*"[Title/Abstract] OR "machine learning*"[Title/Abstract] OR "learning* machine*"[Title/Abstract] OR "transfer learning*"[Title/Abstract] OR "deep learning*"[Title/Abstract] OR "hierarchical learning*"[Title/Abstract] OR "learning from labeled data"[Title/Abstract] OR "supervised* learning*"[Title/Abstract] OR "logistic model*"[Title/Abstract] OR "logistic regression*"[Title/Abstract] OR "logit model*"[Title/Abstract] OR "support vector machine*"[Title/Abstract] OR "support vector network*"[Title/Abstract] OR "comput* neural network*"[Title/Abstract] OR "neural network model*"[Title/Abstract] OR "connectionist model*"[Title/Abstract] OR "perceptron*"[Title/Abstract] OR "computer heuristic*"[Title/Abstract] OR "expert system*"[Title/Abstract] OR "fuzzy logic*"[Title/Abstract] OR "fuzzy cognitive*"[Title/Abstract] OR "decision tree*"[Title/Abstract] OR "random forest*"[Title/Abstract] OR "bayesian approach*"[Title/Abstract] OR "bayes theorem*"[Title/Abstract] OR "bayesian analysis*"[Title/Abstract] OR "bayesian forecast*"[Title/Abstract] OR "bayesian method*"[Title/Abstract] OR "bayesian prediction*"[Title/Abstract] OR "bayesian estimation*"[Title/Abstract] | 754,604 |
|  | #3 #1 OR #2 | 955,742 |
|  | #4 "pregnant women"[MeSH Terms] OR "pregnancy"[MeSH Terms] | 1,042,983 |
|  | #5 "pregnan*"[Title/Abstract] OR "gestation*"[Title/Abstract] | 768,248 |
|  | #6 #4 OR #5 | 1,256,754 |
|  | #7 "infant"[MeSH Terms] | 1,288,734 |
|  | #8 "neonat*"[Title/Abstract] OR "newborn*"[Title/Abstract] OR "infant*"[Title/Abstract] OR "baby"[Title/Abstract] OR "babies"[Title/Abstract] | 895,538 |
|  | #9 #7 OR #8 | 1,639,778 |
|  | #10 "child* under 5"[Title/Abstract] OR "child* younger than 5"[Title/Abstract] OR "child* under five"[Title/Abstract] OR "child* younger than five"[Title/Abstract] OR "under five child*"[Title/Abstract] OR "under 5 child*"[Title/Abstract] | 12,804 |
|  | #11 "mortality"[MeSH Terms] OR "death"[MeSH Terms] OR "survival"[MeSH Terms] | 590,374 |
|  | #12 "mortalit*"[Title/Abstract] OR "death*"[Title/Abstract] OR "fatal outcome*"[Title/Abstract] OR "fatalit*"[Title/Abstract] OR "survival*"[Title/Abstract] OR "end of life"[Title/Abstract] OR "end-of-life"[Title/Abstract] | 2,952,894 |
|  | #13 #11 OR #12 | 3,163,290 |
|  | #14 #6 AND #13 | 158,167 |
|  | #15 #9 AND #13 | 252,777 |
|  | #16 #10 AND #13 | 4,979 |
|  | #17 #14 OR #15 OR #16 | 332,565 |
|  | #18 "sensitivity and specificity"[MeSH Terms] OR "predictive value of tests"[MeSH Terms] OR "roc curve"[MeSH Terms] OR "signal to noise ratio"[MeSH Terms] | 667,253 |
|  | #19 "predictive value*"[Title/Abstract] OR "accuracy"[Title/Abstract] OR "sensitivity"[Title/Abstract] OR "specificity"[Title/Abstract] OR "true positive"[Title/Abstract] OR "true negative"[Title/Abstract] OR "false positive"[Title/Abstract] OR "false negative"[Title/Abstract] OR "ROC"[Title/Abstract] OR "c statistic*"[Title/Abstract] OR "AUROC"[Title/Abstract] OR "AUC"[Title/Abstract] OR "c score*"[Title/Abstract] OR "signal noise*"[Title/Abstract] OR "signal to noise ratio*"[Title/Abstract] OR "o e ratio*"[Title/Abstract] OR "post test probabilit*"[Title/Abstract] OR "likelihood ratio*"[Title/Abstract] OR "predict*"[Title] | 2,446,897 |
|  | #20 #18 OR #19 | 2,749,132 |
|  | #21 #3 AND #17 AND #20 | 3,224 |
| Embase | #1 'artificial intelligence'/exp OR 'machine learning'/exp OR 'deep learning'/exp OR 'supervised machine learning'/exp OR 'unsupervised machine learning'/exp OR 'support vector machine'/exp OR 'logistic regression analysis'/exp OR 'artificial neural network'/exp OR 'computer heuristics'/exp OR 'expert system'/exp OR 'fuzzy logic'/exp OR 'decision tree'/exp OR 'random forest'/exp OR 'bayes theorem'/exp | 769,725 |
|  | #2 'artificial intelligence*':ti,ab,kw OR 'computational intelligence*':ti,ab,kw OR 'machine intelligence*':ti,ab,kw OR 'computer reasoning*':ti,ab,kw OR 'automated reasoning*':ti,ab,kw OR 'computer vision system*':ti,ab,kw OR 'machine learning*':ti,ab,kw OR 'learning* machine*':ti,ab,kw OR 'transfer learning*':ti,ab,kw OR 'deep learning*':ti,ab,kw OR 'hierarchical learning*':ti,ab,kw OR 'learning from labeled data':ti,ab,kw OR 'supervised* learning*':ti,ab,kw OR 'logistic model*':ti,ab,kw OR 'logistic regression*':ti,ab,kw OR 'logit model*':ti,ab,kw OR 'support vector machine*':ti,ab,kw OR 'support vector network*':ti,ab,kw OR 'comput* neural network*':ti,ab,kw OR 'neural network model*':ti,ab,kw OR 'connectionist model*':ti,ab,kw OR 'perceptron*':ti,ab,kw OR 'computer heuristic*':ti,ab,kw OR 'expert system*':ti,ab,kw OR 'fuzzy logic*':ti,ab,kw OR 'fuzzy cognitive*':ti,ab,kw OR 'decision tree*':ti,ab,kw OR 'random forest*':ti,ab,kw OR 'bayesian approach*':ti,ab,kw OR 'bayes theorem*':ti,ab,kw OR 'bayesian analysis*':ti,ab,kw OR 'bayesian forecast*':ti,ab,kw OR 'bayesian method*':ti,ab,kw OR 'bayesian prediction*':ti,ab,kw OR 'bayesian estimation*':ti,ab,kw | 1,003,049 |
|  | #3 #1 OR #2 | 1,345,419 |
|  | #4 'pregnant woman'/exp OR 'pregnancy'/exp | 978,340 |
|  | #5 'pregnan*':ti,ab,kw OR 'gestation*':ti,ab,kw | 1,024,441 |
|  | #6 #4 OR #5 | 1,362,985 |
|  | #7 'newborn'/exp OR 'baby'/exp OR 'infant'/exp | 1,341,879 |
|  | #8 'neonat*':ti,ab,kw OR 'newborn*':ti,ab,kw OR 'infant*':ti,ab,kw OR 'baby':ti,ab,kw OR 'babies':ti,ab,kw | 1,114,688 |
|  | #9 #7 OR #8 | 1,768,494 |
|  | #10 'child* under 5':ti,ab,kw OR 'child* younger than 5':ti,ab,kw OR 'child* under five':ti,ab,kw OR 'child* younger than five':ti,ab,kw OR 'under five child*':ti,ab,kw OR 'under 5 child*':ti,ab,kw | 16,433 |
|  | #11 'mortality'/exp OR 'mortality rate'/exp OR 'death'/exp OR 'survival'/exp OR 'fatality'/exp | 3,349,302 |
|  | #12 'mortalit*':ti,ab,kw OR 'death*':ti,ab,kw OR 'fatal outcome*':ti,ab,kw OR 'fatalit*':ti,ab,kw OR 'survival*':ti,ab,kw OR 'end of life':ti,ab,kw OR 'end-of-life':ti,ab,kw | 4,272,818 |
|  | #13 #11 OR #12 | 5,150,896 |
|  | #14 #6 AND #13 | 210,643 |
|  | #15 #9 AND #13 | 313,153 |
|  | #16 #10 AND #13 | 6,475 |
|  | #17 #14 OR #15 OR #16 | 431,093 |
|  | #18 'sensitivity and specificity'/exp OR 'receiver operating characteristic'/exp OR 'predictive value'/exp OR 'signal noise'/exp | 882,842 |
|  | #19 'predictive value*':ti,ab,kw OR 'accuracy':ti,ab,kw OR 'sensitivity':ti,ab,kw OR 'specificity':ti,ab,kw OR 'true positive':ti,ab,kw OR 'true negative':ti,ab,kw OR 'false positive':ti,ab,kw OR 'false negative':ti,ab,kw OR 'roc':ti,ab,kw OR 'c statistic*':ti,ab,kw OR 'auroc':ti,ab,kw OR 'auc':ti,ab,kw OR 'c score*':ti,ab,kw OR 'signal noise*':ti,ab,kw OR 'signal to noise ratio*':ti,ab,kw OR 'o e ratio*':ti,ab,kw OR 'post test probabilit*':ti,ab,kw OR 'likelihood ratio*':ti,ab,kw OR 'predict*':ti | 3,193,118 |
|  | #20 #18 OR #19 | 3,441,162 |
|  | #21 #3 AND #17 AND #20 | 4,214 |
| Web of Science | #1 TS=("artificial intelligence*" OR "machine learning*" OR "deep learning*" OR "hierarchical learning*" OR "supervised* learning*" OR "support vector machine*" OR "neural network*" OR "expert system*" OR "fuzzy cognitive*" OR "decision tree*" OR "random forest*") | 3,594,012 |
|  | #2 TS=("pregnan*" OR "gestation*" OR "neonat*" OR "newborn*" OR "infant*" OR "baby" OR "babies" OR "child* under 5" OR "child younger than 5" OR "children younger than 5" OR "child* under five" OR "child* younger than five" OR "under five child*" OR "under 5 child*") | 3,963,089 |
|  | #3 TI=("mortalit*" OR "death*" OR "fatal outcome*" OR "fatalit*" OR "survival*") | 1,009,192 |
|  | #4 #2 AND #3 | 86,294 |
|  | #5 (TS=("predictive value*" OR "accuracy" OR "sensitivity" OR "specificity" OR "true positive" OR "true negative" OR "false positive" OR "false negative" OR "ROC" OR "c statistic*" OR "AUROC" OR "AUC" OR "c score*" OR "signal noise*" OR "signal to noise ratio*" OR "o e ratio*" OR "post test probabilit*" OR "likelihood ratio* ")) OR TI=("predict*") | 11,194,097 |
|  | #6 #1 AND #4 AND #5 | 223 |
| Scopus | #1 TITLE-ABS-KEY ("artificial intelligence*" OR "machine learning*" OR "deep learning*" OR "hierarchical learning*" OR "supervised* learning*" OR "support vector machine*" OR "comput* neural network*" OR "expert system*" OR "fuzzy cognitive*" OR "decision tree*" OR "random forest*") | 1,806,601 |
|  | #2 TITLE-ABS-KEY ("pregnan*" OR "gestation*" OR "neonat*" OR "newborn*" OR "infant*" OR "baby" OR "babies" OR "child* under 5" OR "child* younger than 5" OR "child* under five" OR "child* younger than five" OR "under five child*" OR "under 5 child*") | 3,080,873 |
|  | #3 TITLE-ABS-KEY ("mortalit*" OR "death*" OR "fatal outcome*" OR "fatalit*" OR "survival*") | 4,910,184 |
|  | #4 #2 AND #3 | 467,689 |
|  | #5 TITLE-ABS-KEY ("predictive value*" OR "accuracy" OR "sensitivity" OR "specificity" OR "true positive" OR "true negative" OR "false positive" OR "false negative" OR "ROC" OR "c statistic*" OR "AUROC" OR "AUC" OR "c score*" OR "signal noise*" OR "signal to noise ratio*" OR "o e ratio*" OR "post test probabilit*" OR "likelihood ratio*" OR "predict*") | 11,577,106 |
|  | #6 #1 AND #4 AND #5 | 1,721 |
| IEEE Xplore | #1 "All Metadata":"artificial intelligence" OR "All Metadata":"machine learning" OR "All Metadata":"deep learning" OR "All Metadata":"hierarchical learning" OR "All Metadata":"supervised learning" OR "All Metadata":"support vector machine" OR "All Metadata":"neural network" OR "All Metadata":"expert system" OR "All Metadata":"fuzzy cognitive" OR "All Metadata":"decision tree" OR "All Metadata":"random forest" | 860,810 |
|  | #2 "All Metadata":"pregnan*" OR "All Metadata":"gestation*" OR "All Metadata":"neonate" OR "All Metadata":"newborn*" OR "All Metadata":"infant" OR "All Metadata":"infants" OR "All Metadata":"baby" OR "All Metadata":"babies" OR "All Metadata":"child under 5" OR "All Metadata":"children under 5" OR "All Metadata":"child younger than 5" OR "All Metadata":"children younger than 5" OR "All Metadata":"child under five" OR "All Metadata":"children under five" OR "All Metadata":"child* younger than five" OR "All Metadata":"under five child*" OR "All Metadata":"under 5 child*" | 10,803 |
|  | #3 "All Metadata":"mortality" OR "All Metadata":"mortalities" OR "All Metadata":"death" OR "All Metadata":"deaths" OR "All Metadata":"fatal outcome*" OR "All Metadata":"fatality" OR "All Metadata":"fatalities" OR "All Metadata":"survival" | 38,749 |
|  | #4 #2 AND #3 | 1910 |
|  | #5 "Full Text & Metadata":"predictive value" OR "Full Text & Metadata":"accuracy" OR "Full Text & Metadata":"sensitivity" OR "Full Text & Metadata":"specificity" OR "Full Text & Metadata":"true positive" OR "Full Text & Metadata":"true negative" OR "Full Text & Metadata":"false positive" OR "Full Text & Metadata":"false negative" OR "Full Text & Metadata":"ROC" OR "Full Text & Metadata":"c statistic" OR "Full Text & Metadata":"AUROC" OR "Full Text & Metadata":"AUC" OR "Full Text & Metadata":"c score" OR "Full Text & Metadata":"signal noise" OR "Full Text & Metadata":"signal to noise ratio" OR "Full Text & Metadata":"o e ratio" OR "Full Text & Metadata":"post test probability" OR "Full Text & Metadata":"likelihood ratio" | 2,666,068 |
|  | #6 #1 AND #4 AND #5 | 484 |
| Association for Computing Machinery Digital Library | #1 [Abstract: "artificial intelligence*"] OR [Abstract: "machine learning*"] OR [Abstract: "deep learning*"] OR [Abstract: "hierarchical learning*"] OR [Abstract: "supervised* learning*"] OR [Abstract: "support vector machine*"] OR [Abstract: "comput* neural network*"] OR [Abstract: "expert system*"] OR [Abstract: "fuzzy cognitive*"] OR [Abstract: "decision tree*"] OR [Abstract: "random forest*"] | 189,812 |
|  | #2 [Abstract: "pregnan*"] OR [Abstract: "gestation*"] OR [Abstract: "neonat*"] OR [Abstract: "newborn*"] OR [Abstract: "infant*"] OR [Abstract: "baby"] OR [Abstract: "babies"] OR [Abstract: "child* under 5"] OR [Abstract: "child* younger than 5"] OR [Abstract: "child* under five"] OR [Abstract: "child* younger than five"] OR [Abstract: "under five child*"] OR [Abstract: "under 5 child*"] | 2,442 |
|  | #3 [Abstract: "mortalit*"] OR [Abstract: "death*"] OR [Abstract: "fatal outcome*"] OR [Abstract: "fatalit*"] OR [Abstract: "survival*"] | 13,596 |
|  | #4 [Full Text: "predictive value*"] OR [Full Text: "accuracy"] OR [Full Text: "sensitivity"] OR [Full Text: "specificity"] OR [Full Text: "true positive"] OR [Full Text: "true negative"] OR [Full Text: "false positive"] OR [Full Text: "false negative"] | 813,742 |
|  | #5 #2 AND #3 AND #1 AND #4 | 16 |
| CNKI | #1 SU = "人工智能" + "机器学习" + "深度学习" + "监督式学习" + "监督学习" + "支持向量机" + "神经网络" + "专家系统" + "模糊逻辑" + "决策树" + "随机森林" + "贝叶斯算法" + "计算智能" + "自动推理" + "机器推理" + "支持向量回归" | 484,713 |
|  | #2 SU = "孕妇" + "妊娠" + "孕期" + "孕早期" + "孕中期" + "孕晚期" + "怀孕" + "产妇" + "孕产妇" + "围产期" + "产前" + "产中" + "产后" + "分娩" | 519,351 |
|  | #3 SU = "婴儿" + "婴幼儿" + "新生儿" + "初生儿" + "足月儿" | 274,516 |
|  | #4 SU = "5岁以下儿童" + "学龄前" + "学前儿童" | 18,544 |
|  | #5 #2 OR #3 OR #4 | 747,701 |
|  | #6 SU = "死亡率" + "死亡结局" + "生存率" + "存活率" | 168,193 |
|  | #7 #5 AND #6 | 15,921 |
|  | #8 SU = "预测" + "预测值" + "ROC曲线" + "灵敏度" + "特异度" + "真阳性" + "假阴性" + "假阳性" + "真阴性" + "召回率" | 633,199 |
|  | #9 #1 AND #7 AND #8 | 4 |
| WanFang | #1 主题:("人工智能" OR "机器学习" OR "深度学习" OR "监督式学习" OR "监督学习" OR "支持向量机" OR "神经网络" OR "专家系统" OR "模糊逻辑" OR "决策树" OR "随机森林" OR "贝叶斯算法" OR "计算智能" OR "自动推理" OR "机器推理" OR "支持向量回归") | 527,420 |
|  | #2 主题:("孕妇" OR "妊娠" OR "孕期" OR "孕早期" OR "孕中期" OR "孕晚期" OR "怀孕" OR "产妇" OR "孕产妇" OR "围产期" OR "产前" OR "产中" OR "产后" OR "分娩" OR "婴儿" OR "婴幼儿"OR "新生儿" OR "初生儿" OR "足月儿" OR "5岁以下儿童" OR "学龄前" OR "学前儿童") | 1,180,793 |
|  | #3 主题:("死亡率" OR "死亡结局" OR "生存率" OR "存活率") | 454,895 |
|  | #4 #2 AND #3 | 41,380 |
|  | #5 主题:("预测" OR "预测值" OR "ROC曲线" OR "灵敏度" OR "特异度" OR "真阳性" OR "假阴性" OR "假阳性" OR "真阴性" OR "召回率") | 1,216,466 |
|  | #6 #1 AND #4 AND #5 | 8 |
| VIP | #1 M="人工智能" OR M="机器学习" OR M="深度学习" OR M="监督式学习" OR M="监督学习" OR M="支持向量机" OR M="神经网络" OR M="专家系统" OR M="模糊逻辑" OR M="决策树" OR M="随机森林" OR M="贝叶斯算法" OR M="计算智能" OR M="自动推理" OR M="机器推理" OR M="支持向量回归" | 405,038 |
|  | #2 M="孕妇" OR M="妊娠" OR M="孕期" OR M="孕早期" OR M="孕中期" OR M="孕晚期" OR M="怀孕" OR M="产妇" OR M="孕产妇" OR M="围产期" OR M="产前" OR M="产中" OR M="产后" OR M="分娩" | 428,025 |
|  | #3 M="婴儿" OR M="婴幼儿" OR M="新生儿" OR M="初生儿" OR M="足月儿" | 248,356 |
|  | #4 M="5岁以下儿童" OR M="学龄前" OR M="学前儿童" | 20,839 |
|  | #5 #2 OR #3 OR #4 | 674,809 |
|  | #6 M="死亡率" OR M="死亡结局" OR M="生存率" OR M="存活率" | 65,789 |
|  | #7 #5 AND #6 | 7,742 |
|  | #8 M="预测" OR M="预测值" OR M="ROC曲线" OR M="灵敏度" OR M="特异度" OR M="真阳性" OR M="假阴性" OR M="假阳性" OR M="真阴性" OR M="召回率" | 418,620 |
|  | #9 #1 AND #7 AND #8 | 3 |
| SinoMed | #1 "人工智能"[不加权:扩展] OR "机器学习"[不加权:扩展] OR "Logistic模型"[不加权:扩展] OR "神经网络, 计算机"[不加权:扩展] OR "专家系统"[不加权:扩展] OR "模糊逻辑"[不加权:扩展] OR "决策树"[不加权:扩展] OR "贝叶斯定理"[不加权:扩展] | 90,400 |
|  | #2 "人工智能"[常用字段:智能] OR "机器智能"[常用字段:智能] OR "计算机推理"[常用字段:智能] OR "机器学习"[常用字段:智能] OR "深度学习"[常用字段:智能] OR "有监督机器学习"[常用字段:智能] OR "监督学习"[常用字段:智能] OR "Logistic模型"[常用字段:智能] OR "Logit模型"[常用字段:智能] OR "支持向量机"[常用字段:智能] OR "神经网络"[常用字段:智能] OR "专家系统"[常用字段:智能] OR "模糊逻辑"[常用字段:智能] OR "决策树"[常用字段:智能] OR "随机森林"[常用字段:智能] OR "贝叶斯定理"[常用字段:智能] OR "贝叶斯预测"[常用字段:智能] | 102,781 |
|  | #3 #1 OR #2 | 102,781 |
|  | #4 "孕妇"[不加权:扩展] OR "妊娠"[不加权:扩展] OR "围产期"[不加权:扩展] OR "分娩"[不加权:扩展] OR "婴儿"[不加权:扩展] OR "婴儿, 新生"[不加权:扩展] OR "儿童, 学龄前"[不加权:扩展] | 183,309 |
|  | #5 "孕妇"[常用字段:智能] OR "怀孕"[常用字段:智能] OR "妊娠"[常用字段:智能] OR "妊娠末期"[常用字段:智能] OR "围产期"[常用字段:智能] OR "产前"[常用字段:智能] OR "产中"[常用字段:智能] OR "产后"[常用字段:智能] OR "分娩"[常用字段:智能] OR "婴儿"[常用字段:智能] OR "新生儿"[常用字段:智能] OR "婴幼儿"[常用字段:智能] OR "初生儿"[常用字段:智能] OR "足月儿"[常用字段:智能] OR "5岁以下儿童"[常用字段:智能] OR "学龄前儿童"[常用字段:智能] OR "学前儿童"[常用字段:智能] | 789,306 |
|  | #6 #4 OR #5 | 789,306 |
|  | #7 "死亡率"[不加权:扩展] OR "存活率"[不加权:扩展] | 118,813 |
|  | #8 "死亡率"[常用字段:智能] OR "死亡结局"[常用字段:智能] OR "生存率"[常用字段:智能] OR "存活率"[常用字段:智能] | 490,068 |
|  | #9 #7 OR #8 | 490,068 |
|  | #10 #6 AND #9 | 43,793 |
|  | #11 "预测"[不加权:扩展] OR "敏感性与特异性"[不加权:扩展] | 120,087 |
|  | #12 "预测"[常用字段:智能] OR "预测值"[常用字段:智能] OR "ROC曲线"[常用字段:智能] OR "灵敏度"[常用字段:智能] OR "特异度"[常用字段:智能] OR "真阳性"[常用字段:智能] OR "假阴性"[常用字段:智能] OR "假阳性"[常用字段:智能] OR "真阴性"[常用字段:智能] OR "召回率"[常用字段:智能] | 462,141 |
|  | #13 #11 OR #12 | 510,575 |
|  | #14 #3 AND #10 AND #13 | 62 |
| ProQuest Dissertations &Theses Database | #1 Title ("artificial intelligence*" OR "machine learning*" OR "deep learning*" OR "hierarchical learning*" OR "supervised* learning*" OR "support vector machine*" OR "comput* neural network*" OR "expert system*" OR "fuzzy cognitive*" OR "decision tree*" OR "random forest*" OR "bayesian analysis*" OR "bayesian forecast*" OR "bayesian prediction*") | 285,580 |
|  | #2 Title (("pregnan*" OR "gestation*" OR "neonat*" OR "newborn*" OR "infant*" OR "baby" OR "babies" OR "child* under 5" OR "child* younger than 5" OR "child* under five" OR "child* younger than five" OR "under five child*" OR "under 5 child*") AND ("mortalit*" OR "death*" OR "fatal outcome*" OR "fatalit*" OR "survival*")) | 27,159 |
|  | #3 Fulltext ("predictive value" OR "accuracy" OR "sensitivity" OR "specificity" OR "true positive" OR "true negative" OR "false positive" OR "false negative" OR "ROC" OR "c statistic" OR "AUROC" OR "AUC" OR "c score" OR "signal noise" OR "signal to noise ratio*" OR "o e ratio" OR "post test probability" OR "likelihood ratio") | 33,794,101 |
|  | #4 #1 AND #2 AND #3 | 30 |
| CNKI dissertation database | #1 SU = "人工智能" + "机器学习" + "深度学习" + "监督式学习" + "监督学习" + "支持向量机" + "神经网络" + "专家系统" + "模糊逻辑" + "决策树" + "随机森林" + "贝叶斯算法" + "计算智能" + "自动推理" + "机器推理" + "支持向量回归" | 338,193 |
|  | #2 SU = "孕妇" + "妊娠" + "孕期" + "孕早期" + "孕中期" + "孕晚期" + "怀孕" + "产妇" + "孕产妇" + "围产期" + "产前" + "产中" + "产后" + "分娩" | 27,259 |
|  | #3 SU = "婴儿" + "婴幼儿" + "新生儿" + "初生儿" + "足月儿" | 14,366 |
|  | #4 SU = "5岁以下儿童" + "学龄前" + "学前儿童" | 4,187 |
|  | #5 #2 OR #3 OR #4 | 40,759 |
|  | #6 SU = "死亡率" + "死亡结局" + "生存率" + "存活率" | 22,160 |
|  | #7 #5 AND #6 | 627 |
|  | #8 SU = "预测" + "预测值" + "ROC曲线" + "灵敏度" + "特异度" + "真阳性" + "假阴性" + "假阳性" + "真阴性" + "召回率" | 329,588 |
|  | #9 #1 AND #7 AND #8 | 12 |
| WanFang dissertation database | #1 主题:("人工智能" OR "机器学习" OR "深度学习" OR "监督式学习" OR "监督学习" OR "支持向量机" OR "神经网络" OR "专家系统" OR "模糊逻辑" OR "决策树" OR "随机森林" OR "贝叶斯算法" OR "计算智能" OR "自动推理" OR "机器推理" OR "支持向量回归") | 287,628 |
|  | #2 主题:("孕妇" OR "妊娠" OR "孕期" OR "孕早期" OR "孕中期" OR "孕晚期" OR "怀孕" OR "产妇" OR "孕产妇" OR "围产期" OR "产前" OR "产中" OR "产后" OR "分娩" OR "婴儿" OR "婴幼儿"OR "新生儿" OR "初生儿" OR "足月儿" OR "5岁以下儿童" OR "学龄前" OR "学前儿童") | 67,384 |
|  | #3 主题:("死亡率" OR "死亡结局" OR "生存率" OR "存活率") | 118,755 |
|  | #4 #2 AND #3 | 5,256 |
|  | #5 主题:("预测" OR "预测值" OR "ROC曲线" OR "灵敏度" OR "特异度" OR "真阳性" OR "假阴性" OR "假阳性" OR "真阴性" OR "召回率") | 594,914 |
|  | #6 #1 AND #4 AND #5 | 41 |

**Total Results (Search Date: From Inception to 11^st^ October 2024)**

| **Database** | **Results** |
| --- | --- |
| 1. PubMed | n=3,224 |
| 2. Embase | n=4,214 |
| 3. Web of Science | n=223 |
| 4. Scopus | n=1,721 |
| 5. IEEE Xplore | N=484 |
| 6. Association for Computing Machinery Digital Library | n=16 |
| 7. CNKI | n=4 |
| 8. WanFang | n=8 |
| 9. VIP | n=3 |
| 10. SinoMed | n=62 |
| 11. ProQuest Dissertations &Theses Database | n=30 |
| 12. CNKI dissertation database | n=12 |
| 13. WanFang dissertation database | n=41 |
| Total | N=10,042 |
